# New Evidence on the Reliable Use of Stable Isotopes of Bitumen Fractions in Archaeological Research

**DOI:** 10.3390/molecules28041962

**Published:** 2023-02-18

**Authors:** Antonio Pennetta, Giuseppe E. De Benedetto

**Affiliations:** Laboratorio di Spettrometria di Massa Analitica e Isotopica, Dipartimento di Beni Culturali, Università del Salento, 73100 Lecce, Italy

**Keywords:** stable isotopes, bitumen, EA-IRMS, lipid absorption, asphaltene

## Abstract

One of the goals of archaeological studies is to determine how material goods and ideas moved among human populations, and bitumen is a worthy proxy because it has been used since prehistory. As a result, when bitumen is excavated from archaeological sites, determining its provenance is important because it sheds light on the trade and communication of populations at a given time. During the study of archaeological bitumen from coastal sites in central and southern Puglia (Italy), we observed that stable isotope ratios of saturated and aromatic fractions were incompatible with those obtained from asphaltenes, supporting the absorption of a foreign substance. Experiments showed that lipids are absorbed by bitumen and, in the case of oils, are distributed mainly in the saturated and aromatic fractions as their isotopic ratios change. The same experiments showed that the isotopic ratios of the asphaltenes do not change. Lipid absorption on the archaeological bitumen may have occurred before the bitumen was applied to the pottery, during the use of the pottery or while underground, before being excavated. These hypotheses are discussed, and it is concluded that the isotopic ratio of asphaltenes is a reliable proxy for provenance, whereas those of the saturated and aromatic fractions should be considered with caution due to possible lipid absorption. Nevertheless, they provide new information on pottery use that can be used in archaeological chemistry.

## 1. Introduction

Recent results have pushed back the first known date for the use of natural bitumen by early hominids to about 70,000 BP and show that older Middle Paleolithic populations were already aware of the adhesive properties of bitumen and used it as hafting material to glue handles onto their tools [1,2]. Thus, the provenance of bitumen provides important clues to trade and communication from the Middle Paleolithic onwards. Since then, the use of bitumen has spread throughout the Middle East, Egypt and the Mediterranean as an adhesive for repairing pottery, a pigment for decoration, a sealant for containers, a fuel for torches, a chemical for embalming, a sealant for boat caulking, etc. [3,4,5].

Bitumen is a complex viscoelastic substance composed of thousands of hydrocarbon molecules of different molecular mass. The dichloromethane soluble organic matter, which represents what is commonly called bitumen, can be fractionated into four organic fractions: saturates, aromatics, resins and asphaltenes. From a chemical point of view, the saturated fraction comprises acyclic alkanes and alkenes, and mono- to hexa-ringed cycloalkanes: within this fraction there are many biomarkers used in the chemical fingerprinting of bitumen [6] such as tricyclic terpanes, steranes (four rings) and hopanes (five rings). Aromatic hydrocarbons have one or more benzene rings and obey the formula C_n_H_2n−6y_, where y is the number of aromatic rings. Aromatic hydrocarbons can be divided into classes based on the number of aromatic rings. Alkylated benzenes, naphthalenes and phenanthrenes are the most abundant aromatic species. Triaromatic steroids, highly resistant to biodegradation, are often used as a correlation parameter [6]. Resins and asphaltenes have different solubilities in hydrocarbons: the former are dissolved by low-molecular-weight alkanes (e.g., *n*-hexane) whereas the latter are not. This difference in behavior is the result of a rough distinction in molecular dimension, not a net distinction in chemical class: both fractions are not a specifically defined class of compounds but mixtures of high-molecular-weight heteroatomic molecules [6]. The stable isotope ratios of the saturated, aromatic, resin and asphaltene fractions are naturally correlated with those of the corresponding bitumen: these fractions are, indeed, enriched in δ^13^C with increasing polarity and boiling point, and differences between the saturated and asphaltene fractions, the least and most enriched in δ^13^C, respectively, are less than 1‰ [6,7].

As documented in all previous studies of archaeological bitumen since the pioneering works of Connan [3,8,9], molecular tools, especially sterane and terpane distribution patterns, have been used to determine the origin of the bitumen [1,9,10,11,12,13,14,15,16,17,18,19,20,21,22]. Bulk stable carbon and deuterium isotope analyses [23,24], and the isotopic ratio of saturated, aromatic and asphaltene fractions [2,8,12,16,25,26,27,28,29,30], have also been used for bitumen provenance.

The excavations carried out at Roca (Figure S1) have brought to light an impressive system of fortifications dating back to the Bronze Age (XV-XI centuries BC), as well as numerous finds that recall Minoan and Aegean models. It is believed that it was an important place of worship, and in the temple hut, numerous vases were found, even of large size, in which olive oil was stored [31,32]. Grotta dei Cervi is a natural coastal cave in Porto Badisco (Figure S1). It is a remarkable Neolithic pictorial complex in Europe, full of pictograms in bat guano and red ochre, representing hunters, animals, magical symbols and abstract geometries. There is also one of the most famous pictograms in the world, the so-called Dancing God, which depicts a dancing shaman. It was frequented mainly between the first half of the sixth millennium BC and the last centuries of the second millennium BC [33]. More than thirty thousand finds have been excavated and archeozoological studies have shown the presence of both terrestrial and marine faunal remains [33]. At both archaeological sites, some of the pottery shards had black residue on them, which we examined and identified as bitumen [34,35]. Moreover, thanks to the comparison with geological bitumen collected in different seepages, pits and mines in the central Mediterranean area, it was found that the bitumen originated from mines located near Selenice (Figure S1) in Albania [34,35]. The origin of the samples was assessed on the basis of various biomarker ratios, some of them being C_24_ tricyclic terpane/C_23_ tricyclic terpane, C_24_ tetracyclic terpane/C_23_ tricyclic terpane, T_s_/T_m_, gammacerane/17α(H),21β(H)-30-norhopane; C_33_ isohopanes/regular C_33_ hopanes, C_33_ 30-norhopanes/regular C_33_ hopanes and %C_27_ steranes/(C_27_ + C_28_ + C_29_ steranes). These results were not unexpected since all the bitumen samples from other archaeological sites along the central and southern Apulian coast, such as Monopoli and Torre Santa Sabina (Figure S1), had the same provenance [11]. The carbon stable isotope ratios of the asphaltene fraction of the Porto Badisco (PB) [34] and Roca (RO) [35] samples are −29.2‰ (±0.1‰) and −29.3‰(±0.1‰), respectively, in the same range as the asphaltenes of the Selenice bitumen (−29.1 ± 0.1‰) [34]. In geological bitumen, the saturated and aromatic fractions are enriched in ^13^C by less than 1‰ relative to the asphaltenes [6,7]. The aims of this study are to investigate whether this is also true for the archaeological bitumen samples and to test a model relating the isotopic composition of the bitumen fractions to the bulk isotopic ratio of foreign substances that may have contaminated the bitumen during its use, or the use of the vessels on which it was applied, up to the time of the shard excavation. The results have potentially important implications for a broader understanding of bitumen provenance and pottery use in archaeological research.

## 2. Results

Five black residues scraped from ceramics unearthed at Grotta dei Cervi (Porto Badisco, PB) and five from Roca (RO) were recently analyzed [34,35]. Ceramics from Grotta dei Cervi are dated from an advanced phase of the Early Neolithic (first half of the sixth millennium BC) [33], whereas those from Roca are from the end of the Bronze Age (last centuries of the second millennium BC) [31,32]. Three out of five samples from PB and four out of five from RO were actually bitumen, and its source could be identified thanks to the molecular sterane and terpane parameters and the carbon and sulfur stable isotope analysis of the asphaltene (δ^13^C, δ^34^S) [34,35]: all the bitumen had the same origin, Selenice in Albania, which is on the other side of the Adriatic Sea at about 100 km from Roca and Porto Badisco (Figure S1).

**Figure 1:**
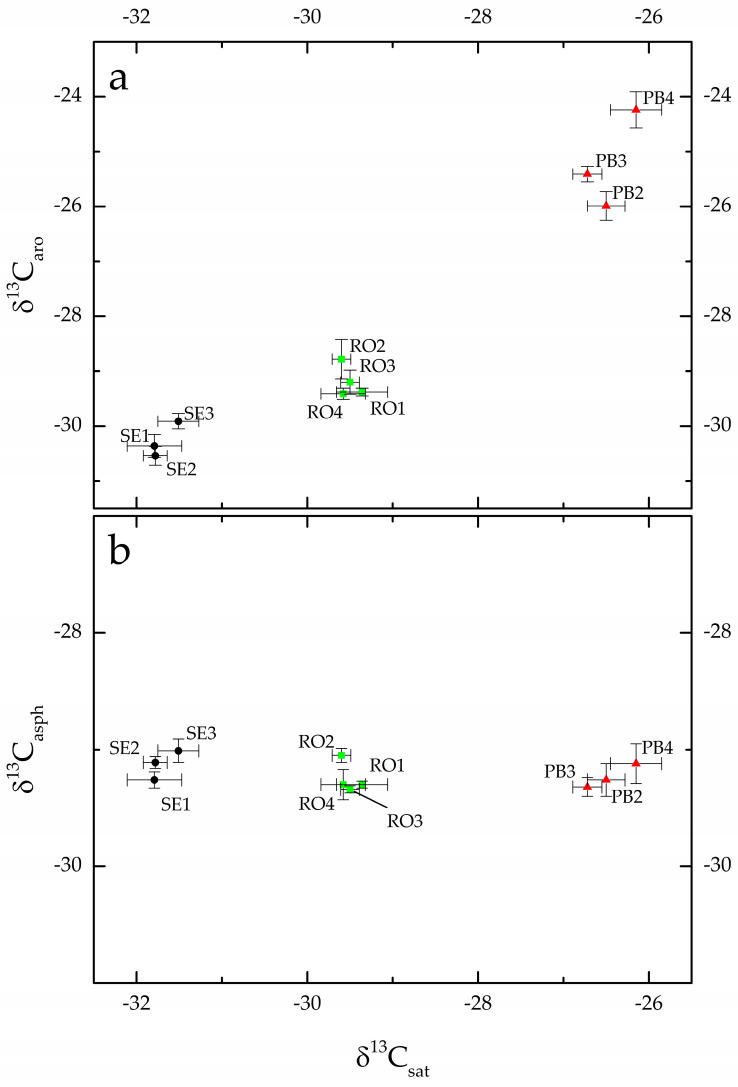
(**a**) δ^13^C_aro_ vs. δ^13^C_sat_ and (**b**) δ^13^C_asph_ vs. δ^13^C_sat_ graphs for archaeological samples from Roca (RO) and Porto Badisco (PB) and Selenice geological bitumen (SE). δ^13^C_asph_ data of SE and PB samples are from reference [34], whereas δ^13^C_asph_ data of RO samples are from reference [35].

In the past and recent literature [12,28,29], δ^13^C_sat_ and δ^13^C_aro_ have also been used to find the provenance of archaeological bitumen samples. These measurements were performed on PB, RO and SE samples: Table 1 lists both δ^13^C_sat_ and δ^13^C_aro_ and Figure 1a shows the resulting graph.

The mean saturated isotopic ratio of Porto Badisco samples was 26.5‰, whereas the mean aromatic isotopic ratio was 25.2‰. These values are different from those recorded for all the geological bitumen samples from Selenice, which averaged −31.7 and −30.3‰, respectively. The bitumen samples from Roca also have saturated and aromatic fractions that are enriched in ^13^C: the average δ^13^C_sat_ and δ^13^C_aro_ of the four samples were −29.5 and −29.2‰. Figure 1b compares the carbon isotope ratios of the saturated and asphaltene fractions. This heavier and more polar fraction exhibits isotopic ratios equal to −29.2‰, −29.3‰ and −29.1‰ for the PB [34], RO [35] and SE [34] bitumen samples, respectively.

The isotopic ratios of the different bitumen fractions are strictly related and have increasing δ^13^C with increasing polarity and boiling point, but differences are not greater than 1‰ [6,7]. Hence, the results shown in Figure 1a,b suggest that the saturated and aromatic fractions of the archaeological samples were contaminated with a foreign substance.

Hints on the nature of this foreign substance can be obtained considering the bitumen fractionation procedure: the first step was the dissolution of the sample in dichloromethane (DCM) for the separation of the extractable organic matter (EOM) from the solid residues. DCM can dissolve a wide range of organic compounds, both polar and apolar: it was used, in fact, as an extractant for naturally occurring, heat-sensitive substances such as caffeine, edible fats, hops, cocoa and spices. Then asphaltenes were recovered as residue by treating EOM with a large volume of *n*-hexane, which dissolves aliphatic and aromatic hydrocarbon and resins. Finally, the saturated and aromatic fractions were separated by column chromatography according to polarity on activated silica. Different classes of compounds can be dissolved by DCM; however, only lipids are dissolved by *n*-hexane, so contamination by a lipid substance is supported.

The differences recorded between the isotopic ratios of the archaeological and geological samples (Figure 1a) suggest that C_3_ vegetable oils or ruminant fats could be the lipids absorbed by RO samples, whereas fish or porcine fats could be those absorbed by PB samples. For experimental ease, olive oil (δ^13^C_bulk_ = −30.07‰) and fish oil (δ^13^C_bulk_ = −23.01‰) were selected as substances with different isotopic ratios to verify the capability of bitumen to absorb lipids and how lipid molecules distribute themselves among asphaltenes and saturated and aromatic fractions. Six pieces of SE2 bitumen, which was the source of the archaeological samples according to previous works [34,35], were soaked for 6 h in the fish (n = 3) and olive (n = 3) oils. Then the pieces were removed from oil, washed with distilled water, dried and fractionated: asphaltene, saturated and aromatic fractions were collected and analyzed by EA-IRMS. Table 2 lists the measured stable isotope ratios.

**Figure 2:**
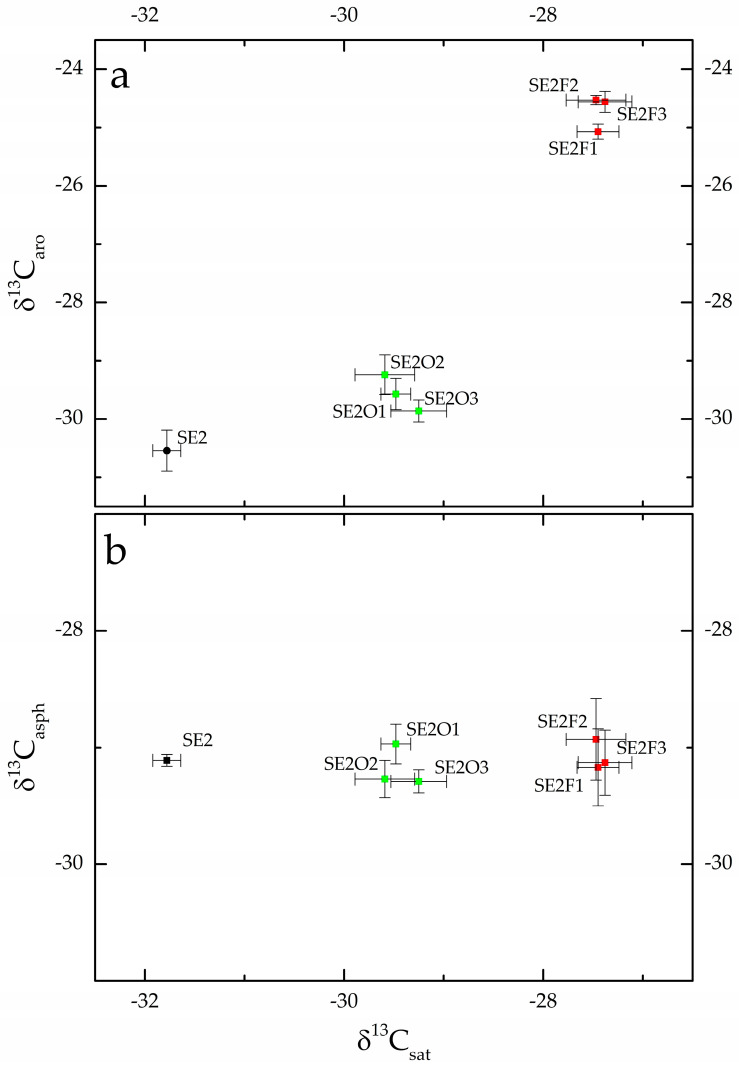
(**a**) δ^13^C_aro_ vs. δ^13^C_sat_ and (**b**) δ^13^C_asph_ vs. δ^13^C_sat_ graph of both geological SE2 bitumen and of the SE2 bitumen soaked in olive (SE2O) and fish (SE2F) oils.

The saturated fractions of SE2 bitumen soaked in olive oil (SE2O) exhibit an average isotopic ratio of −29.5‰, while those soaked in fish oil exhibit an average isotopic ratio of −27.5‰. During fractioning, lipids also partition into the aromatic fractions: the aromatic fractions of SE2 bitumen soaked in oils are enriched in ^13^C as δ^13^C of the SE2O samples averages −29.6 and δ^13^C of the SE2F samples averages −24.7‰. This is illustrated by the graph of δ^13^C_sat_ vs. δ^13^C_aro_ in Figure 2a. Both the saturated and aromatic fractions of bitumen soaked in oils (SE2O and SE2F) are, in fact, enriched in ^13^C with respect to SE2 geological bitumen, and SE2F samples are further apart according to the different δ^13^C of the fish oil. The average δ^13^C_sat_ and δ^13^C_aro_ of the RO samples are −29.5 and −29.2‰, within the same range of the averages recorded for the SE2O samples, −29.5 and −29.6‰, respectively: these data suggest that olive oil may be the lipid absorbed by the archaeological samples, but due to the very complex chemistry of bitumen, it was not possible to support this hypothesis with molecular evidence. Figure 2b shows the graph δ^13^C_asph_ vs. δ^13^C_sat_: δ^13^C_asph_ of the SE2O and SE2F samples average −29.2 and −29.1‰, respectively, and these values are not different from the value recorded for the geological sample from Selenice.

Py-GCMS is a common tool used to analyze polymeric materials with no or little preparation. Both SE2 and RO2 samples have been analyzed by py-GCMS and their pyrograms are shown in Figure S2: the profiles of archaeological and geological bitumen are very similar, and the main peaks in the geological sample are also present in the archaeological one and vice versa. *n*-alkanes and 1-alkenes, which are the main pyrolysis products of triglycerides, have been identified, but it is not possible to assess the contribution of foreign lipids in archaeological samples because they are also formed during the pyrolysis of geological bitumen. Interestingly, the pyrogram of the archaeological sample also shows the presence of both methyl and dimethylthiophenes (Figure S3) whose boiling points are of about 115 and 135 °C [36].

## 3. Discussion

Bitumen on potsherds from the archaeological sites of Roca and Porto Badisco was studied using molecular and stable isotope analyses [34,35]. The sterane (*m*/*z* 217) and terpane (*m*/*z* 191) distribution patterns from Porto Badisco and Roca are characterized by regular steranes C27-C29 in a “V” pattern with C29 predominance, the presence of diasteranes, low amounts of tricyclopolyprenanes compared to the dominant αβ-hopane family, the prevalence of 17α(H),21β(H)-30-norhopane with respect to 17α(H),21β-hopane, low gammacerane and the presence of isohomohopanes and 30-norhopanes (Table 2 in [34] and Table 1 in [35]). Only one sample (PB3) lost some of its regular C27-C29 steranes, resulting in a higher diasterane/sterane ratio (Table 2 in [34]), but these differences are mainly due to different degrees of biodegradation/weathering that affected the molecular distribution pattern. In addition, the carbon and sulfur stable isotope ratios of the asphaltenes are within the same range in all of these archaeological bitumen samples [34,35]. The examination of the 25 biomarker distributions of the saturated fractions and the isotopic data of the asphaltenes of the Porto Badisco and Roca samples definitely shows that the analyzed archaeological bitumen has a unique source. Based on the available sources analyzed, the bitumen was imported from the Selenice mine in Albania [34,35]. This mine is on the other side of the Adriatic Sea, about 100 km air-line distance from the archaeological sites (Figure S1). During these studies, differences were observed between the isotopic ratios of the saturated and aromatic fractions of the archaeological samples and the geological sample of origin (Figure 1a and Table 1) that could not be explained and deserved consideration.

Stable isotope analysis has emerged in the last decades as one of the most powerful tools for tracing organic carbon in food webs, and, even if the variable environmental conditions influence carbon isotopic ratios [37], δ^13^C remains useful, for instance, for the distinction of aquatic and terrestrial sources. “Marine” carbon in fact comes from dissolved inorganic carbon (dissolved bicarbonate) characterized by an isotopic value of about 0‰; hence, higher than that of atmospheric carbon dioxide, which is around −7‰. This difference is preserved at every trophic level in both the marine and terrestrial environments. Stable isotopes are also used in organic residue analysis: C3 vegetable oils, such as olive oil, have δ^13^C of about −30‰ [38], a value not different to those recorded for ruminant fats [39], whereas porcine fats are enriched in ^13^C up to about −25‰, a value similar to those reported for saltwater fish [39]. Moreover, when substances with different isotopic ratios are mixed, the isotopic ratios of the mixtures exhibit intermediate values [40], and these patterns are well known in organic residue analysis [40,41,42]. As a result, Figure 1a can be explained by a mixture of the geological bitumen with foreign substances exhibiting different isotopic ratios. In addition, the archaeological samples are clustered according to their origin, so that accidental contamination can be excluded: the Selenice bitumen at Porto Badisco was mixed with other substances more enriched in ^13^C compared to those used at Roca. Olive oil and fish oil were chosen because of their different isotopic ratios and the simplicity of the experiment. However, the data reported in Table 1 (and shown in Figure 1a) were not collected on the bulk of the bitumen, so it was necessary to verify how these oils were partitioned into saturated, aromatic and asphaltene fractions. The experimental results in Figure 2a show that the saturated and aromatic fractions are mixtures of molecules from the absorbed lipids with, respectively, the saturated and aromatic hydrocarbons from bitumen. Conversely, Figure 2b shows that all the asphaltene fractions exhibit isotopic ratios within the same range, thus demonstrating that during bitumen fractionation, the absorbed lipids are mainly partitioned into saturated and aromatic fractions and not into the asphaltenes.

These results show, for the first time to our knowledge, that bitumen absorbs lipids and this evidence, which has no implications for the study of geological bitumen, must be considered when studying archaeological remains.

Considering the life cycle of the sample from bitumen collection from geological deposits to its application on pottery, pottery use and disposal, to final excavation, this absorption could have occurred during the pretreatment of the bitumen prior to its use on ceramics, during the use of the pottery or while underground, before being excavated.

As to the first hypothesis, bitumen often requires a pretreatment step [40] to transform it into a mastic (processed bitumen), consisting of mixing the raw bitumen with inorganic (such as limestone, sand, ash, etc.) or organic, generally fibrous (such as minced straw or papyrus) materials: this processed bitumen had the consistency of rock asphalt but a lower melting temperature [40]. Selenice bitumen is solid and brittle and, as with rock asphalt from outcrops, it has a higher melting temperature than liquid or semisolid bitumen, such as those from the Tramutola seepage (Basilicata, Italy) [41] or the Herodotus spring (Zakynthos, Greece) [4,42]. Its softening temperature range is composed of two different phases, one is at a lower temperature, due to the presence of the lighter maltenes, whereas the second one, at a higher temperature, is caused by asphaltenes: at an increasing temperature and before melting, when it has an elastic behavior, it becomes workable [4,40]. Selenice bitumen has a melting temperature of 105–115 °C [43,44], so it could be heated at about 120–140 °C and used before it cools without any pretreatment. This straightforward use of Selenice bitumen has some supporting evidence: the absence of organic and inorganic additives in all the archaeological bitumen of Albanian origin [11,34], the similar profiles of archaeological and geological bitumen pyrograms (Figure S2), and the presence of methylthiophenes and dimethylthiophenes in the pyrograms of the archaeological samples (Figure S3). These molecules indicate that archaeological bitumen samples, either before or after being applied to pottery, were not heated directly in a fire. In fact, when a vessel is directly set on fire, the temperature rises above 250–300 °C and all substances with a lower boiling temperature, such as methyl and dimethyl thiophenes, evaporate. Temperature control is obviously necessary: a water bath is not sufficient to reach the melting temperature of Selenice bitumen, so it is very likely that it was achieved by the use of cooking plates. Cooking plates that could have been used for this purpose, in fact, have been discovered in Monopoli and Torre Santa Sabina [45] where the use of Selenice bitumen was demonstrated [11].

Lipid absorption during pottery use was demonstrated by soaking bitumen in oils. Interestingly, saturated and aromatic fractions were contaminated, whereas asphaltenes were not. This is not surprising as vegetable oils, such as olive oil, are mixtures of triglycerides (mainly), fatty acids, tocopherols, sterols, polyphenols, pigments, hydrocarbons, aromatic and aliphatic alcohols, triterpene acids and waxes. Within each organism, isotopic compositions of these classes cluster tightly because they are related to the same source [46], i.e., produced using the same organic carbon that is generated by a specific biochemical pathway (autotrophs) or ingested with food (heterotrophs). Fish oil is much richer in polyunsaturated fatty acids, but the same classes of compounds are present. All these classes are well soluble in n-hexane: considering that asphaltenes are not soluble in *n*-hexane, unlike saturated, aromatic and resin fractions, it is clear why they are not contaminated with oils.

All archaeological bitumen samples could have absorbed lipids from their contents: from one side in those samples (PB2, PB4, RO2 and RO4) where the bitumen has been used to repair broken sherds, or from the whole surface in the case of PB3, RO1 and RO3 where the bitumen was used to impermeabilize the ceramic.

The hypothesis of lipid absorption by the bitumen while underground, before being excavated, cannot be excluded because of the presence in the soils of long-chain alkanes and alcohols, waxy esters and humic acids [47,48]. The total lipid extracts of soil samples are generally dominated by the contribution of leaf-derived lipids [47], whose stable carbon isotope ratios depend on the biochemical pathway used to fix carbon dioxide. C_3_ plants dominate the regions with a Mediterranean climate characterized by a moist cool winter and dry warm summer [49,50], so the differences in δ^13^C_sat_ and δ^13^C_aro_ of the RO and PB samples cannot be justified by the different C_4_/C_3_ contributions in primary production because the sites are only 40 km apart. In fact, millets (*Panicum miliaceum* and/or *Setaria italica*), which are C_4_ plants, first occurred in Northern Italy, following their introduction from across the Alps in Central Europe, during the Bronze age, and are not attested in Southern Italy during the first half of the sixth millennium BC [51].

Although bitumen contamination either before its application to pottery or while underground cannot be ruled out, it is most likely that bitumen absorbed lipids from food during the pottery’s life. This hypothesis, taking into account the isotopic values measured and the fact that all the Roca samples have similar isotopic ratios, but different from those of Porto Badisco, implies that the latter were mainly contaminated with fish or pig fats, while the Roca samples were contaminated with vegetable oils or ruminant fats. The number of samples analyzed is small and more samples are desirable, but the data support the absorption of lipids by bitumen. It is also noteworthy that the δ^13^C of saturated and aromatic fractions is distinctly modified, while the δ^13^C of asphaltenes is always within the same range. Thus, the carbon isotope ratio of asphaltene is a more reliable parameter of the original bitumen [21,22] and represents a more consistent proxy to be used for its provenance, whereas the hydrocarbon isotopic ratios also possess the signature of pottery use. These findings have potentially important implications, paving the way for new applications in archaeological research.

## 4. Materials and Methods

### 4.1. Samples

Basic information on the archaeological samples excavated at Porto Badisco and Roca including their gross composition, biomarker (steranes and terpanes) ratios and isotopic ratios of asphaltenes, have already been reported [34,35]. The provenance of these archaeological samples was Selenice bitumen mine (40°32′08.2″ N 19°38′42.1″ E). Bitumen from this mine was used for the experiments on lipid absorption. The oils used in these experiments were an olive oil from a local producer (extra virgin olive oil from *Leccino* cultivar, Agricole Negro Valiani, Presicce-Acquarica, Lecce, Italy) and a commercial fish oil (Omega 3 Fish Oil, Nu U Nutrition Ltd., Urmston, Greater Manchester, UK).

Three pieces of the Selenice bitumen having a weight of about 100 mg each were immersed in the olive oil and three in the fish oil. Samples were left in oil about 6 h, then gently removed (pieces are easily dispersed by oil) and leaned on filter paper, washed dropwise with distilled water and finally dried.

### 4.2. Bitumen Fractionation

All archaeological and geological samples were subjected to the same analytical procedure already outlined [11]. In summary, bitumen samples were extracted with dichloromethane (DCM) to separate organic extractable matter from kerogen or inorganic material. The extracts were dried, and *n*-hexane added to separate asphaltenes, which are not soluble, from saturated, aromatic and polar fractions. After centrifugation for 5 min at 2000 rpm, the *n*-hexane extracts were transferred to a clean vial, the precipitates were washed at least three times with fresh *n*-hexane and the washing solutions were added to the extract. The asphaltenes were dried. The extracts, after concentration, were loaded onto silica columns (Supelco, affiliate of Merck KGaA, Darmstadt, Germany, 100 mesh) activated at 400 °C prior to use to separate saturated and aromatic fractions. The saturated fractions were eluted with *n*-hexane, the aromatic ones with *n*-hexane/DCM (70:30, *v*/*v*). Both fractions were then dried for analysis.

### 4.3. EA-IRMS Analyses

The stable carbon isotope ratios (δ^13^C) of the saturated, aromatic and asphaltene fractions were determined using an Elementar IsoPrime 100 isotope-ratio mass spectrometry (IRMS) instrument (IsoPrime Ltd., Cheadle Hulme, UK) coupled to an N-C-S elemental analyzer (Pyro Cube EA CNS; Elementar Analysensysteme GmbH, Hanau, Germany). The sample (ca. 0.01 mg) was weighted with a microbalance (±0.001 mg) Sartorius CP2 P-F (Sartorius AG, Goettingen, Germany), loaded and sealed in a tin capsule. Samples were placed in an autosampler mounted on the Pyro Cube EA. Combustion was carried out in tube heated at 1020 °C with oxygen at a gas flow of 35 mL/min, whereas helium at a gas flow of 230 mL/min was used as carrier. The produced gases were separated in adsorption columns and desorbed into the mass spectrometer for isotopic analysis. The δ^13^C values were calculated in per mil (‰) relative to the VPDB standard (uncertainty ± 0.1‰) using IAEA–CH–7 polyethylene (–32.151‰) and BRC-657 glucose (−10.76‰) as certified reference materials and CO_2_ at 4 bar as internal reference gas. Data were elaborated with the ionOS software (Elementar Analysensysteme GmbH, Hanau, Germany).

### 4.4. Py-GC-MS

Small amounts (ca. 0.1–0.5 mg) of the sample were inserted into the tube of a ferromagnetic wire. The wire was placed in a glass liner and then introduced in the pyrolysis unit CPP (Pilodist, Bonn, Germany). Pyrolysis conditions were the following: Curie point temperature 500 °C, pyrolysis time 9.9 s, temperature of the pyrolysis transfer line 200 °C. The pyrolysis unit is flushed with helium, which is also the carrier gas, and is mounted directly on the split/splitless injector of a 6890N (Agilent Technologies Italia SpA, Cernusco Sul Naviglio, Italy) gas chromatograph coupled with a 5973inert (Agilent Technologies Italia SpA, Cernusco Sul Naviglio, Italy) mass spectrometer. The injector temperature was 280 °C and pyrolysate was injected in splitless or 1:20 split mode. The solvent delay was 3 min. A Restek RXI-5Sil MS column (L 30 m, i.d. 0.25, 0.25 µm film thickness) was used to separate pyrolysate with the following oven temperature program: 40 °C (2 min), 5 °C/min to 300 °C (6 min). MS transfer line temperature: 300 °C. MS conditions: linear quadrupole, EI ionization 70 eV, cycle time 2.94 scan/s, mass range *m*/*z* 50–550. Data were acquired and elaborated with MS Chemstation (Agilent Technologies Italia SpA, Cernusco Sul Naviglio, Italy). Identification of compounds was performed with the NIST08 MS library search program.

## 5. Conclusions

Archaeological samples from two different archaeological sites showed different isotope ratios for the saturated and aromatic fractions. The possibility of lipid adsorption by bitumen was demonstrated by specific experiments. It was also shown that these foreign lipids do not contaminate asphaltenes during bitumen fractionation into asphaltene, saturated, aromatic and resin fractions. Thus, the asphaltene isotopic ratio is a more reliable proxy for bitumen provenance, while the hydrocarbon isotopic ratios also carry the signature of ceramic use. This model made it possible to highlight the absorption of lipids of different origins for the archaeological samples from Roca and Porto Badisco. Although great care must be taken in interpreting the data, a new possibility in archaeological research has been established. Overall, the results have potentially important implications for bitumen provenance and pottery use.

## Figures and Tables

**Table 1:** Isotope data of archaeological (RO and PB) and geological (SE) bitumen samples; sat: saturated hydrocarbons; aro: aromatic hydrocarbons. Standard deviation of three measurements.

Sample	Label	δ^13^C_sat_. ‰VPDB	δ^13^C_aro_. ‰VPDB
US 113 CASS. 282 H3	RO1	−29.4 ± 0.3	−29.4 ± 0.1
US 503 CASS. 128 H12	RO2	−29.6 ± 0.1	−28.8 ± 0.4
PL12 CASS. 64 L11	RO3	−29.5 ± 0.1	−29.2 ± 0.2
US 5664 CASS. 2269 O15	RO4	−29.6 ± 0.3	−29.4 ± 0.1
GD2	PB2	−26.5 ± 0.2	−26.0 ± 0.3
GD3	PB3	−26.7 ± 0.2	−25.4 ± 0.1
GD4	PB4	−26.2 ± 0.3	−24.2 ± 0.3
Selenice bitumen (40°32′10.6″ N 19°38′41.3″ E)	SE1	−31.8 ± 0.3	−30.4 ± 0.2
Selenice bitumen (40°32′08.2″ N 19°38′42.1″ E)	SE2	−31.8 ± 0.1	−30.5 ± 0.2
Selenice bitumen (40°29′11.6″ N 19°39′00.3″ E)	SE3	−31.5 ± 0.2	−29.9 ± 0.1

**Table 2:** Isotope data of the geological SE2 bitumen used for soaking experiments in fish and olive oils and of the resulting SE2F and SE2O samples; sat: saturated hydrocarbons; aro: aromatic hydrocarbons; asph: asphaltene fraction. Standard deviation of three measurements.

Sample	Label	δ^13^C_sat_. ‰VPDB	δ^13^C_aro_. ‰VPDB	δ^13^C_asph_. ‰VPDB
Selenice bitumen	SE2	− 31.8 ± 0.1	− 30.5 ± 0.2	− 29.1 ± 0.1
SE2 bit. + fish oil	SE2F1	− 27.5 ± 0.2	− 25.1 ± 0.1	− 29.2 ± 0.2
SE2 bit. + fish oil	SE2F2	− 27.5 ± 0.3	− 24.5 ± 0.1	− 28.9 ± 0.2
SE2 bit. + fish oil	SE2F3	− 27.4 ± 0.3	− 24.6 ± 0.2	− 29.1 ± 0.1
SE2 bit. + olive oil	SE2O1	− 29.5 ± 0.2	− 29.6 ± 0.3	− 29.0 ± 0.2
SE2 bit. + olive oil	SE2O2	− 29.6 ± 0.3	− 29.2 ± 0.3	− 29.3 ± 0.2
SE2 bit. + olive oil	SE2O3	− 29.3 ± 0.3	− 29.9 ± 0.2	− 29.3 ± 0.1

## Data Availability

Not applicable.

## Supplementary Materials

The following supporting information can be downloaded at: https://www.mdpi.com/article/10.3390/molecules28041962/s1, Figure S1: Map showing the geographical region in which archaeological and geological sites are located. Figure S2: Pyrograms of (a) the geological bitumen SE2 and (b) the archaeological sample RO2. Figure S3: (a) XIC of the ion 97, characteristic of methyl and dimethyl thiophenes. (b) Mass spectrum of the peak at 10.22 in which 2,4-dimethylthiophene coelutes with o-xylene; (c) mass spectrum of the peak at 10.48 relevant to 2,3-dimethylthiophene; (d) mass spectrum of the peak at 10.80 relevant to 2,5-dimethylthiophene.
