# Supplementary material for: New Evidence on the Reliable Use of Stable Isotopes of Bitumen Fractions in Archaeological Research
Source: Molecules. 2023 Feb 18;28(4):1962. doi: 10.3390/molecules28041962 (PMC9964228; doi:10.3390/molecules28041962)
Supplement: Supplementary file 1 [file molecules-28-01962-s001.zip › molecules-2155784-supplementary.pdf]

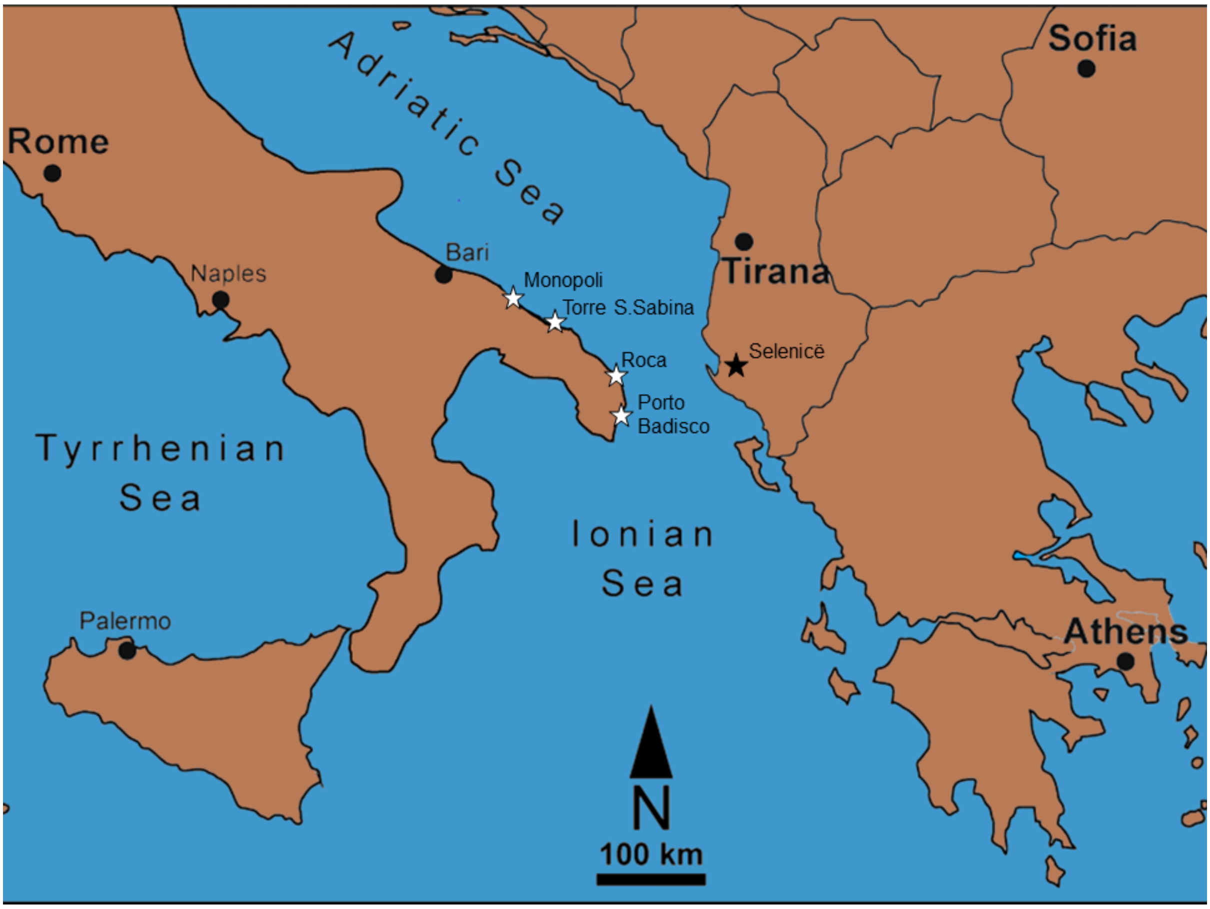

Figure S1. Map of the geographical area where both archaeological (open stars) and geological (full stars) sites are located.

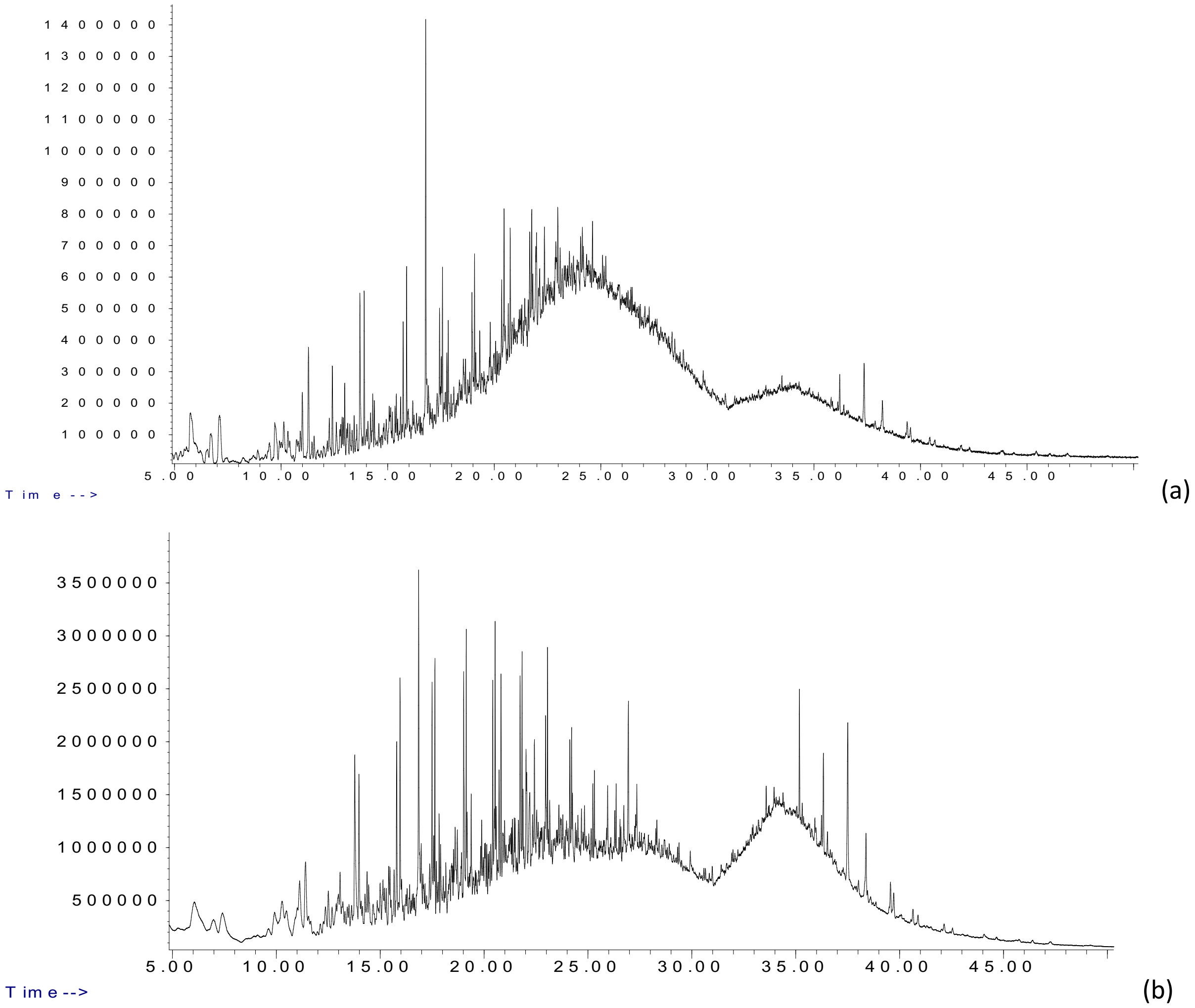

Figure S2. Pyrograms of (a) the geological Selenice bitumen and (b) the archaeological sample RO2

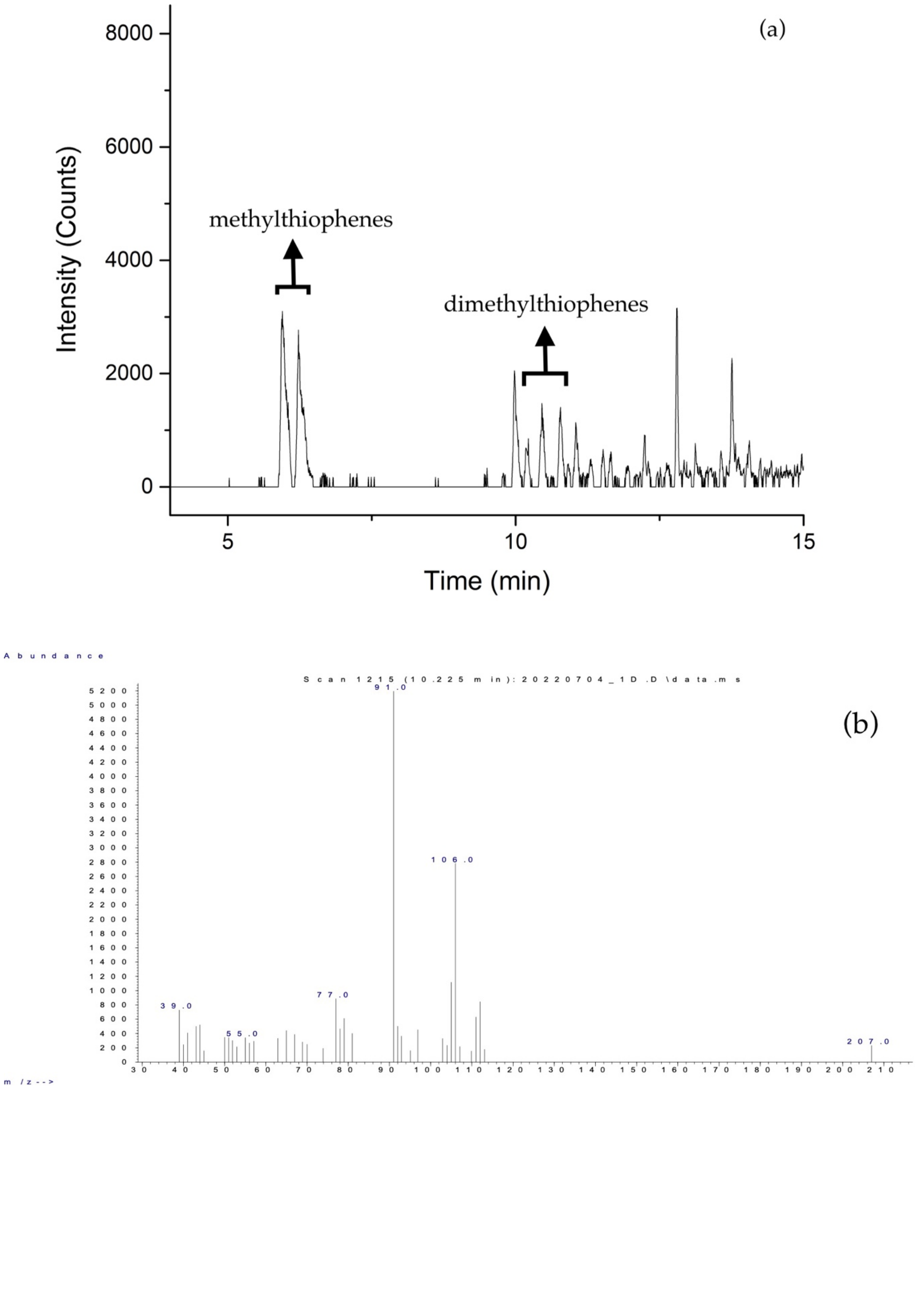

(a)
Intensity (Counts)
Time (min)
methylthiophenes
dimethylthiophenes
0
2000
4000
6000
8000
5
10
15
(b)
Abundance
Scan 1215 (10.225 min): 20220704_1D.D\data.ms
91.0
106.0
77.0
39.0
55.0
207.0
m/z-->

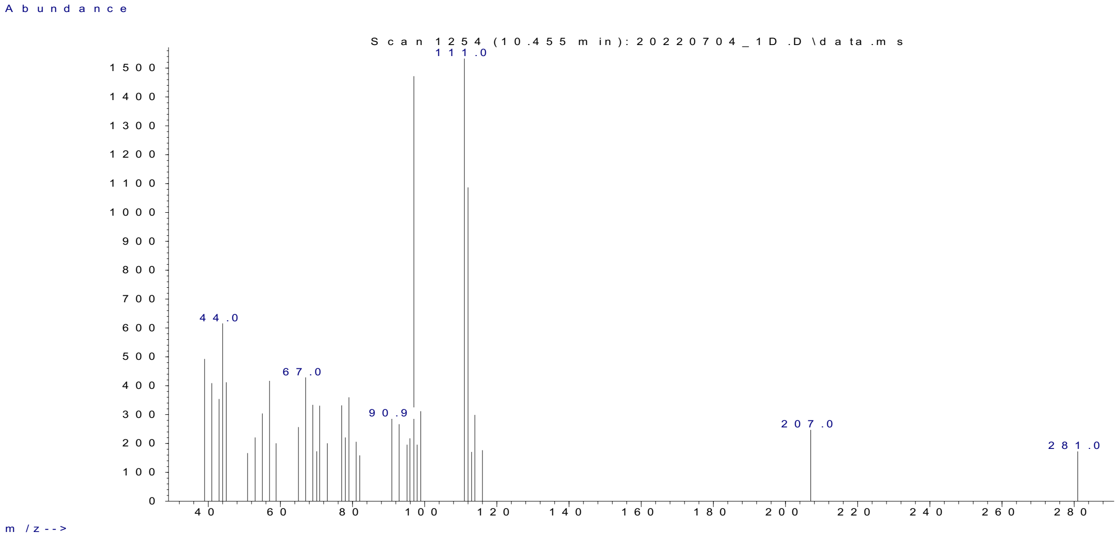

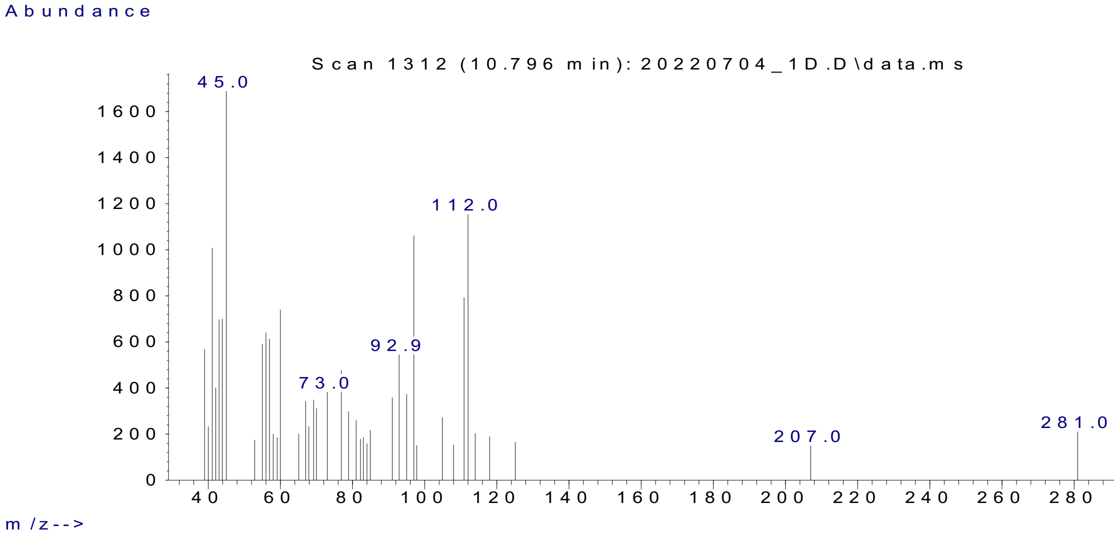

Figure S3. (a) XIC of the ion 97, characteristic of methyl and dimethyl thiophenes. The peaks at 10.22, 10.48 and 10.80 min are relevant to dimethylthiophenes. (b) mass spectrum of the peak at 10.22 in which 2,4-dimethylthiophene coelutes with o-xylene; (c) mass spectrum of the peak at 10.48 relevant to 2,3-dimethylthiophene; (d) mass spectrum of the peak at 10.80 relevant to 2,5-dimethylthiophene.
